# A KSHV microRNA enhances viral latency and induces angiogenesis by targeting GRK2 to activate the CXCR2/AKT pathway

**DOI:** 10.18632/oncotarget.8591

**Published:** 2016-04-05

**Authors:** Wan Li, Xuemei Jia, Chenyou Shen, Mi Zhang, Jingyun Xu, Yuancui Shang, Kaixiang Zhu, Minmin Hu, Qin Yan, Di Qin, Myung-Shin Lee, Jianzhong Zhu, Hongmei Lu, Brian J. Krueger, Rolf Renne, Shou-Jiang Gao, Chun Lu

**Affiliations:** ^1^ State Key Laboratory of Reproductive Medicine, Nanjing Medical University, Nanjing, P. R. China; ^2^ Key Laboratory of Pathogen Biology of Jiangsu Province, Nanjing Medical University, Nanjing, P. R. China; ^3^ Department of Microbiology, Nanjing Medical University, Nanjing, P. R. China; ^4^ Department of Gynecology and Obstetrics, Nanjing Maternity and Child Health Hospital Affiliated Hospital of Nanjing Medical University, Nanjing, P. R. China; ^5^ The Fourth Clinical Medical College of Nanjing Medical University, Nanjing, P. R. China; ^6^ Department of Microbiology and Immunology, Eulji University School of Medicine, Daejeon, Republic of Korea; ^7^ Cancer Virology Program, University of Pittsburgh Cancer Institute, Pittsburgh, PA, USA; ^8^ Department of Obstetrics, The First Affiliated Hospital of Nanjing Medical University, Nanjing, P.R. China; ^9^ Department of Molecular Genetics and Microbiology, University of Florida, Gainesville, FL, USA; ^10^ Department of Molecular Microbiology and Immunology, Keck School of Medicine, University of Southern California, Los Angeles, CA, USA

**Keywords:** KSHV miRNAs, latency, angiogenesis, GRK2, CXCR2

## Abstract

Kaposi's sarcoma-associated herpesvirus (KSHV) is the causative agent of Kaposi's sarcoma (KS), primary effusion lymphoma (PEL) and multicentric Castleman's disease (MCD). Most tumor cells in these malignancies are latently infected by KSHV. Thus, viral latency is critical for the development of tumor and induction of tumor-associated angiogenesis. KSHV encodes more than two dozens of miRNAs but their roles in KSHV-induced angiogenesis remains unknown. We have recently shown that miR-K12-3 (miR-K3) promoted cell migration and invasion by targeting GRK2/CXCR2/AKT signaling (PLoS Pathog, 2015;11(9):e1005171). Here, we further demonstrated a role of miR-K3 and its induced signal pathway in KSHV latency and KSHV-induced angiogenesis. We found that overexpression of miR-K3 not only promoted viral latency by inhibiting viral lytic replication, but also induced angiogenesis. Further, knockdown of GRK2 inhibited KSHV replication and enhanced KSHV-induced angiogenesis by enhancing the CXCR2/AKT signals. As a result, blockage of CXCR2 or AKT increased KSHV replication and decreased angiogenesis induced by PEL cells in vivo. Finally, deletion of miR-K3 from viral genome reduced KSHV-induced angiogenesis and increased KSHV replication. These findings indicate that the miR-K3/GRK2/CXCR2/AKT axis plays an essential role in KSHV-induced angiogenesis and promotes KSHV latency, and thus may be a potential therapeutic target of KSHV-associated malignancies.

## INTRODUCTION

Kaposi's sarcoma-associated herpesvirus (KSHV) is an oncogenic herpesvirus etiologically associated with Kaposi's sarcoma (KS), which is the most common tumor in AIDS patients. KSHV is also associated with two rare lymphoproliferative malignancies known as primary effusion lymphoma (PEL) and multicentric Castleman's disease (MCD) [1]. KS lesions are histologically characterized by abnormally dense and irregular blood vessels, extravasated erythrocytes with hemosiderin deposits, and vast inflammatory infiltration. KS tumor cells expressing several endothelial markers though the origin of these tumor cells remain unclear. Unlike KS, the lymphoma cells are usually originated from pre-B cells, and PEL is characterized as a malignant effusion in the peritoneal, pleural, or pericardial space, usually without a tumor mass; however, some PEL can also present as solid masses [2].

Like other herpesviruses, the lifecycle of KSHV has latent and lytic replication phases [1]. Most tumor cells in KS tumors are latently infected by KSHV and express a limited subset of viral latent genes including latency-associated nuclear antigen (LANA; ORF73), viral cyclin (vCyclin; ORF72), viral FLICE inhibitory protein (vFLIP; ORF71) and a cluster of 12 viral precursor-microRNAs (pre-miRNAs). These latent genes, which are located within the latency-associated region, promote cell growth, survival and the development of KSHV-induced tumors [3–5]. KSHV latent infection is also an effective strategy for evading immune detection [3, 5]. Consequently, the mechanism controlling KSHV latency has been the hot topic in the field in the last decade.

MiRNAs are ~22-nucleotide-long RNAs that typically bind with imperfect complementarity to the 3′ untranslated regions (UTRs) of the target mRNAs and cause translational repression and mRNA degradation. MiRNAs are involved in diverse cellular functions and in all phases of cancer development [6]. Virus-encoded miRNAs, especially the ones belong to herpesvirus family, offers an attractive system to study viral lifecycle and pathogenesis. As a γ2-herpesvirus, KSHV encodes 12 pre-miRNAs genes giving rise to at least 25 mature miRNAs, which are all expressed in latently infected cells and largely unaffected after induction of lytic replication [7–13]. Several KSHV miRNAs stabilize viral latency by directly targeting viral genes or indirectly targeting cellular pathways [14–20]. KSHV miRNAs also regulate diverse cellular pathways, which might contribute to the development of KSHV-related malignancies [15, 21–38].

Among them, miR-K3 has been shown to maintain viral latency by controlling nuclear factor I/B (NFIB), which could activate the promoter of the viral immediate-early *trans*activator replication and transcription activator (RTA) [19]. Furthermore, miR-K3 contributes to the maintenance of latency by decreasing RTA expression via down-regulation of MYB, C/EBPα and Ets-1 [39]. We have recently revealed that miR-K3 directly targets G protein-coupled receptor kinase 2 (GRK2) to promote endothelial cell migration and invasion by activating CXCR2/AKT signaling [40].

Due to the high expression levels of miR-K3 in KS lesions [41] and the crucial role that the miR-K3/GRK2/CXCR2/AKT axis might play in the KS pathogenesis [40], in this study, we have shown that, by targeting GRK2, miR-K3 not only facilitates KSHV latency, but also mediates KSHV-induced angiogenesis by activating the CXCR2/AKT pathway. These novel findings demonstrate that by targeting the GRK2/CXCR2/AKT pathway through encoding a miRNA, KSHV maintains its latency and induces a pro-angiogenic microenvironment to promote tumorigenesis. This work provides further insights into the roles of viral miRNAs in the pathogenesis of KSHV-related tumors.

## RESULTS

### GRK2/CXCR2/AKT signaling is altered in B lymphoma cells latently infected by KSHV

**Figure 1:**
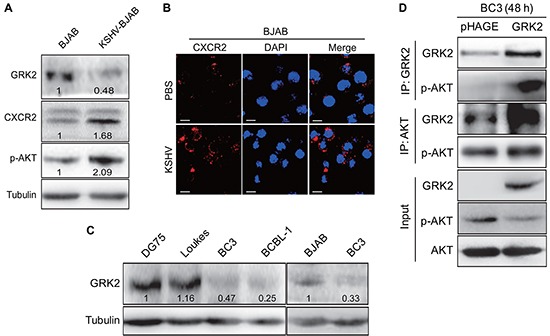
The GRK2/CXCR2/AKT pathway is changed in B lymphoma cells infected by KSHV **A.** Western blotting analysis of GRK2, CXCR2 and phosphorylated AKT in KSHV latently infected BJAB cells (KSHV-BJAB) and uninfected control cells (BJAB). Numbers labeled under the bands were the relative intensities of the bands following calibration for loading by house-keeping proteins. Some of the following Western blotting figures have similar densitometry analysis. **B.** IFA for the expression of CXCR2 (red) in BJAB cells infected with KSHV (KSHV; bottom) or treated with PBS (PBS; top). DAPI (blue) stains nuclei (Original magnifications, x1000). **C.** Western blotting analysis of GRK2 in KSHV-infected PEL cells (BC3 and BCBL-1 cells) and KSHV-negative lymphoma cells (DG75, Loukes, and BJAB cells), respectively. **D.** Co-immunoprecipitation of GRK2 and AKT in GRK2-overexpressing BC3. BC3 cells were transduced with lentivirus-GRK2 (GRK2) or its control (pHAGE) for 48 h, respectively, and then subjected to immunoprecipitation with antibody against GRK2 (IP: GRK2) or AKT (IP: AKT). At the same time, cell lysates were immunoblotted with indicated antibodies (Input).

We have recently shown that KSHV miR-K3 hijacks the GRK2/CXCR2/AKT signaling pathway to promote migration and invasion of endothelial cells [40]. To determine whether this pathway was also altered in KSHV-infected B cells, Western blotting was performed in BJAB cells latently infected by KSHV [42]. As shown in Figure 1A, KSHV infection of BJAB cells inhibited the expression of GRK2 and increased the levels of CXCR2 and phosphorylated AKT. Consistently, immunofluorescence assay (IFA) showed more CXCR2-positive cells in KSHV-infected BJAB cells than those uninfected BJAB cells (Figure 1B). Examination of PEL cell lines showed the expression of GRK2 was in general lower in KSHV-positive PEL cells including BC3 and BCBL-1 cells than in KSHV-negative lymphoma cells including DG75, Loukes and BJAB cells (Figure 1C). Given our finding that GRK2 directly bound to AKT to inhibit its function in endothelial cells [40], we investigated the interaction between GRK2 and AKT in BC3 cells. The results from co-immunoprecipitation assay showed that overexpression of GRK2 by transduction of lentivirus-GRK2 led to more phosphorylated AKT bound to GRK2 but decreased level of activated AKT in the cell lysate (Figure 1D).

### MiR-K3 promotes KSHV latency and angiogenesis

**Figure 2:**
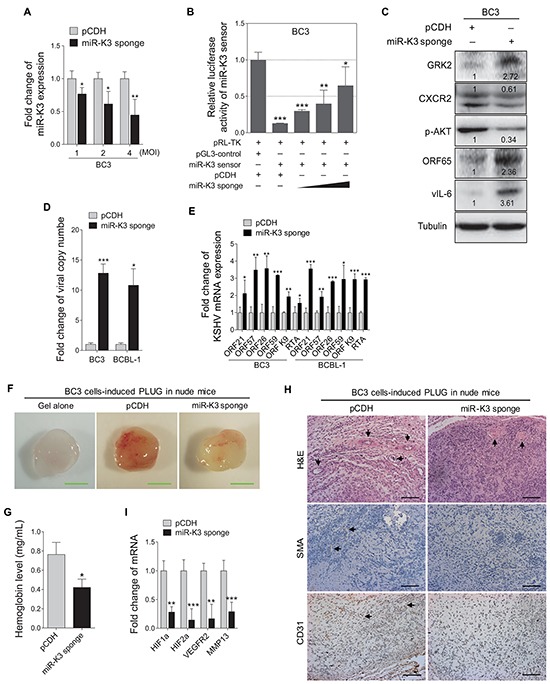
Inhibition of miR-K3 in KSHV latently infected PEL cells induces KSHV lytic replication and inhibits angiogenesis **A.** RT-qPCR for detection of miR-K3 expression in BC3 cells transduced by lentivirus-mediated miR-K3 sponge. BC3 cells were transduced with an increasing MOI of lentivirus-mediated miR-K3 sponge (miR-K3 sponge) or its control (pCDH). *P < 0.05 and **P < 0.01 for Student's *t*-test. **B.** MiR-K3 sponge was functional. BC3 cells were co-transfected with miR-K3 sensor reporter and pRL-TK, and subsequently transduced with an increasing MOI of lentivirus-mediated miR-K3 sponge (miR-K3 sponge) or its control (pCDH). The cells were collected at 48 h post-transduction for luciferase assays. *P < 0.05, **P < 0.01 and ***P < 0.001 for Student's *t*-test. **C.** Inhibition of miR-K3 altered the GRK2/CXCR2/AKT pathway and increased the levels of KSHV lytic proteins. BC3 cells were transduced with lentivirus-mediated miR-K3 sponge (miR-K3 sponge) or its control (pCDH) for 48 h and then analyzed by Western blotting for the protein levels of GRK2, CXCR2, phosphorylated AKT, ORF65 and vIL-6. **D.** Real-time DNA-PCR analysis for the viral genome copy number in the supernatant of cells treated as in (C). *P < 0.05 and ***P < 0.001 for Student's *t*-test. **E.** RT-qPCR analysis for the mRNA expressions of viral lytic genes in cells treated as in (C). ORF21, ORF57, ORF26, ORF59, ORF-K9 and RTA were included. *P < 0.05, **P < 0.01 and ***P < 0.001 for Student's *t*-test. **F.** Inhibition of miR-K3 reduced KSHV-induced angiogenesis in nude mice. BC3 cells were transduced with miR-K3 sponge (miR-K3 sponge) or the control (pCDH) for 48 h. As described in the ‘Materials and Methods’ section, the treated cells were injected (s.c.) into nude mice for 10 days to detect the pro-angiogenic effects in the Matrigel plug assay. Representative photographs of angiogenesis in nude mice were shown. **G.** The hemoglobin level of the Matrigel plugs treated as in (**F**) was determined with O.D. value at 540 nm in BC3 cells-induced tumor tissues. Data represented mean ± SD, each group with five tumors (*n*=5). Three independent experiments were performed and similar results were obtained. *P < 0.05 for Student's *t*-test. **H.** Hematoxylin and eosin (H&E) staining of histologic features (top; original magnification, x200) and immunohistochemical staining analysis (IHC) of SMA (middle; original magnification, x200) and CD31 (bottom; original magnification, x200) in tumor tissues from the Matrigel plugs treated as in (F). Black arrows pointed to neovascularization and hemorrihagic foci in H&E stained sections, SMA and CD31 in IHC sections, respectively. **I.** RT-qPCR analysis of the mRNA expressions of HIF1α, HIF2α, VEGFR2 and MMP13 in the tumor tissues from the Matrigel plugs treated as in (F). **P < 0.01 and ***P < 0.001 for Student's *t*-test.

MiR-K3 has been reported to maintain viral latency by targeting nuclear factor I/B, MYB, C/EBPα and Ets-1 [19, 39]. In addition, we have found that miR-K3 directly targets GRK2/CXCR2/AKT pathway to promote migration and invasion of endothelial cells [40]. However, whether GRK2/CXCR2/AKT pathway is also involved in miR-K3 regulation of viral latency remains unknown. The miR-K3 sponge construct, which has been proved functional in KSHV-infected HUVEC [40], was utilized to inhibit the function of miR-K3 in KSHV latently infected B cells. RT-qPCR showed that transduction of lentivirus-mediated miR-K3 sponge in BC3 dramatically reduced the detectable level of miR-K3 (Figure 2A). In the luciferase reporter assay, transduction of the sponge abolished the inhibitory effect of miR-K3 on its sensor reporter in a dose-dependent manner in BC3, indicating that the miR-K3 sponge effectively inhibited the endogenous miR-K3 function in BC3 (Figure 2B). Further, the expression of GRK2 was increased and the levels of CXCR2 and phosphorylated AKT were significantly down-regulated in BC3 cells transduced with the miR-K3 sponge (Figure 2C).

To determine if inhibition of miR-K3 affect KSHV latency, we examined the expression of KSHV lytic proteins. Transduction with the miR-K3 sponge increased the expression of small capsid protein encoded by ORF65 and viral interleukin-6 (vIL-6) (Figure 2C). The results of real-time PCR for KSHV DNA indicated that repression of miR-K3 by the miR-K3 sponge increased KSHV genome copy number in the supernatants of BC3 and BCBL-1 cells (Figure 2D), as well as the expression of ORF21, ORF57, ORF26, ORF59, ORF-K9 and RTA transcripts in both cells (Figure 2E). Together, these results indicated that inhibition of miR-K3 disrupt KSHV latency and induced KSHV lytic replication program. Thus, miR-K3 might promote KSHV latency.

Angiogenesis, referred to as the formation of new blood vessels from existing vasculature, plays a key role in tumor growth and progression. Since miR-K3 promoted KSHV latency, and some latent genes or viral miRNAs such as LANA and miR-K6-5p were located in KSHV latency locus, involved in angiogenesis [22, 43], and actively transcribed during latency, we hypothesized that miR-K3 could also mediate KSHV-induced angiogenesis. While PEL is an effusion lymphoma, it is often involved with solid tumor [44–51]. The development and progression of these solid tumors require tumor angiogenesis. Therefore, we decided to test the role of miR-K3 in PEL-induced angiogenesis. Matrigel plug assay was performed in nude mice. As expected, BC3 cells induced strong angiogenic effects (Figure 2F–2H). By detecting the hemoglobin content in the plug, which represented the relative angiogenesis index, we showed that inhibition of miR-K3 expression decreased the angiogenic capability of BC3 cells by Matrigel plug assay (Figure 2F and 2G). Meanwhile, the results from hematoxylin and eosin (H&E) and immunohistochemical staining (IHC) showed less erythrocyte infiltration in blood vessels and the lower expression of smooth muscle actin (SMA) and CD31 in tumors derived from the Matrigel plugs treated with miR-K3 sponge compared with those of controls (Figure 2H). Consistently, suppression of miR-K3 also decreased the transcript levels of several angiogenic factors, including HIF1α, HIF2α, and VEGFR2, and invasion-related factor, MMP13 (Figure 2I). Considering that B cells facilitate new blood vessels formation through recruiting host endothelial cell, we analyzed the amounts of angiogenic chemokines or cytokines in the supernatants. Indeed, inhibition of miR-K3 reduced the secretion of VEGFA protein to the supernatant of BC3 cells (Supplementary Figure S1A).

**Figure 3:**
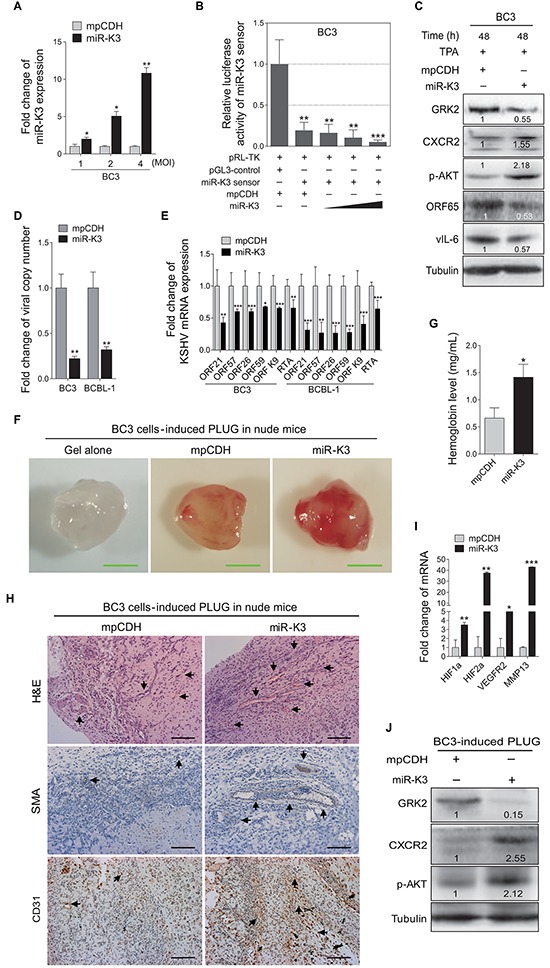
Overexpression of miR-K3 in KSHV latently infected cells inhibits KSHV lytic replication and promotes angiogenesis **A.** RT-qPCR for detection of miR-K3 expression in BC3 cells transduced by lentivirus-mediated miR-K3. BC3 cells were transduced with an increasing MOI of lentivirus-mediated miR-K3 (miR-K3) or its control (mpCDH). *P < 0.05 and **P < 0.01 for Student's *t*-test. **B.** MiR-K3 construct was functional. BC3 were co-transfected with miR-K3 sensor reporter and pRL-TK, and subsequently transduced with an increasing MOI of lentivirus-mediated miR-K3 (miR-K3) or its control (mpCDH). The cells were collected at 48 h post-transduction for luciferase assays. **P < 0.01 and ***P < 0.001 for Student's *t*-test. **C.** Overexpression of miR-K3 regulated the GRK2/CXCR2/AKT pathway and decreased the levels of KSHV lytic proteins. BC3 cells were transduced with lentivirus-miR-K3 (miR-K3) or the empty vector (mpCDH) before TPA treatment for 48 h. The transduced cells were analyzed by Western blotting with the indicated antibodies. **D.** Overexpression of miR-K3 significantly impaired the production of KSHV infectious progeny virus. Real-time DNA-PCR was used to detect the viral genome copy number in the supernatant of cells treated as in (C). **P < 0.01 for Student's *t*-test. **E.** Overexpression of miR-K3 inhibited the mRNA transcripts of KSHV lytic genes. RT-qPCR was performed to detect the viral transcript levels in cells treated as in (C), including ORF21, ORF57, ORF26, ORF59, ORF-K9 and RTA. The shown data were from triplicate independent experiments. *P < 0.05, **P < 0.01 and ***P < 0.001 for Student's *t*-test. **F.** Overexpression of miR-K3 enhanced KSHV-induced angiogenesis in nude mice. BC3 cells were transduced with lentivirus-miR-K3 (miR-K3) or the control lentivirus (mpCDH) for 48 h. As described in the ‘Materials and Methods’ section, the treated cells were injected (s.c.) into nude mice for 10 days to detect the pro-angiogenic effects in the Matrigel plug assay. Representative photographs of angiogenesis in nude mice were shown. **G.** The hemoglobin level of the Matrigel plugs treated as in (F) was determined with O.D. value at 540 nm in BC3 cells-induced tumor tissues. Data represented mean ± SD, each group with five tumors (*n*=5). Three independent experiments were performed and similar results were obtained. *P < 0.05 for Student's *t*-test. **H.** H&E staining analysis of histologic features (top; original magnification, x200) and immunohistochemical staining analysis (IHC) of SMA (middle; original magnification, x200) and CD31 (bottom; original magnification, x200) in tumor tissues from the Matrigel plugs treated as in (F). Black arrows pointed to neovascularization and hemorrihagic foci in H&E stained sections, SMA and CD31 in IHC sections, respectively. **I.** RT-qPCR analysis of the transcriptional expression levels of HIF1α, HIF2α, VEGFR2 and MMP13 in the tumor tissues from the Matrigel plugs treated as in (D). *P < 0.05, **P < 0.01 and ***P < 0.001 for Student's *t*-test. **J.** Western blotting analysis of the levels of GRK2, CXCR2 and phosphorylated AKT in the tumor tissues treated as in (F).

To further confirm the above results, we overexpressed miR-K3 in BC3 cells. RT-qPCR showed that lentivirus-mediated miR-K3 overexpression in BC3 increased miR-K3 expression (Figure 3A). In luciferase reporter assay, transduction of miR-K3 in BC3 cells decreased the activities of the miR-K3 sensor reporter in a dose-dependent manner, indicating that the miR-K3 construct was functional (Figure 3B). Western blotting showed that transduction of lentivirus-mediated miR-K3 in BC3 cells markedly inhibited GRK2 expression and increased the levels of CXCR2 and phosphorylated AKT. Importantly, KSHV lytic proteins, including small capsid protein encoded by ORF65 and vIL-6, were dramatically decreased (Figure 3C). Consistently, there were reduced viral particles in the supernatants of BC3 and BCBL-1 cells following miR-K3 overexpression (Figure 3D). The results from qPCR demonstrated that overexpression of miR-K3 inhibited the expression of KSHV lytic transcripts, including ORF21, ORF57, ORF26, ORF59, ORF-K9 and RTA (Figure 3E).

Similarly, in the angiogenesis assay, overexpression of miR-K3 in BC3 cells increased the hemoglobin content (Figure 3F and 3G). H&E staining of the tumors derived from the Matrigel plugs showed that there were more extensive dense neovascularization and hemorrhagic necrotic foci in tumors induced by miR-K3-overexpressed BC3 cells when compared to control. Meanwhile, tumors induced by miR-K3-overexpressed BC3 cells had more SMA and CD31 positive cells (Figure 3H). Consistently, the mRNA expression levels of HIF1α, HIF2α, VEGFR2, and MMP13 were dramatically elevated in tumors with overexpression of miR-K3 (Figure 3I). Western blotting analysis performed with tumors revealed that overexpression of miR-K3 not only reduced the expression of GRK2, but also increased CXCR2 and phosphorylated AKT expression (Figure 3J). Examination of the supernatants showed that there was more secreted VEGFA protein in miR-K3-overexpression BC3 cells (Supplementary Figure S1B).

Taken together, these results indicate that miR-K3 plays a vital role both in KSHV latency and angiogenesis in PEL cells, and the GRK2/CXCR2/AKT pathway regulated by miR-K3 may participate in this process.

### GRK2 negatively regulates miR-K3-induced KSHV latency and angiogenesis

**Figure 4:**
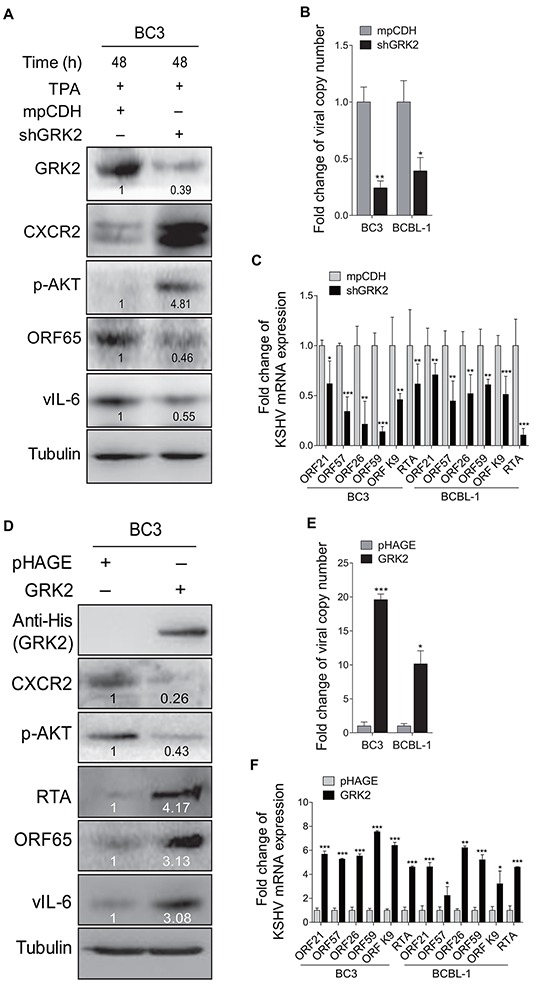
GRK2 contributes to reactivation of KSHV from latency **A.** Western blotting analysis of the expression levels of GRK2, CXCR2, phosphorylated AKT, ORF65 and vIL-6 in BC3 cells transduced with lentivirus-mediated a mixture of short hairpin RNA targeting GRK2 (shGRK2) or the vector control (mpCDH) and stimulated with TPA for 48 h. **B.** Real-time DNA-PCR was used to detect the viral genome copy number in the supernatants of BC3 and BCBL-1 cells treated as in (A). *P < 0.05 and **P < 0.01 for Student's *t*-test. **C.** The mRNA expression levels of ORF21, ORF57, ORF26, ORF59, ORF-K9 and RTA in BC3 and BCBL-1 cells treated as in (A) were quantitated by RT-qPCR. *P < 0.05, **P < 0.01 and *** *P* < 0.001 for Student's *t*-test. **D.** Western blotting analysis of the expression levels of CXCR2, phosphorylated AKT, RTA, ORF65 and vIL-6 in BC3 cells transduced with lentivirus-GRK2 (GRK2) or the control (pHAGE). The antibody against His-tag was used to detect the exogenous expression of GRK2. **E.** Real-time DNA-PCR was used to detect the viral genome copy number in the supernatants of BC3 and BCBL-1 cells treated as in (D). *P < 0.05 and ***P < 0.001 for Student's *t*-test. **F.** The mRNA expression levels of ORF21, ORF57, ORF26, ORF59, ORF-K9 and RTA in BC3 and BCBL-1 cells treated as in (D) were quantitated by RT-qPCR. *P < 0.05, **P < 0.01 and ***P < 0.001 for Student's *t*-test.

To examine the role of GRK2 in miR-K3 regulation of KSHV lifecycle, BC3 cells were transduced with a mixture of lentivirus-mediated short hairpin RNAs targeting GRK2 (shGRK2). As shown in Figure 4A, knockdown of GRK2 elevated the levels of CXCR2 and phosphorylated AKT, and inhibited the protein expression levels of ORF65 and vIL-6. Consistently, inhibition of GRK2 decreased the production of viral particles and the expression of ORF21, ORF57, ORF26, ORF59, ORF-K9 and RTA mRNA levels in BC3 and BCBL-1 cells (Figure 4B and 4C). To confirm these results, we ectopically expressed GRK2 in BC3 and BCBL-1. We found that overexpression of GRK2 reduced the levels of CXCR2 and phosphorylated AKT but increased the expression of viral lytic proteins, including RTA, ORF65 and vIL-6, as well as the copy number of KSHV progeny virions in the culture supernatants and the expression of KSHV lytic transcripts (Figures 4D–4F).

**Figure 5:**
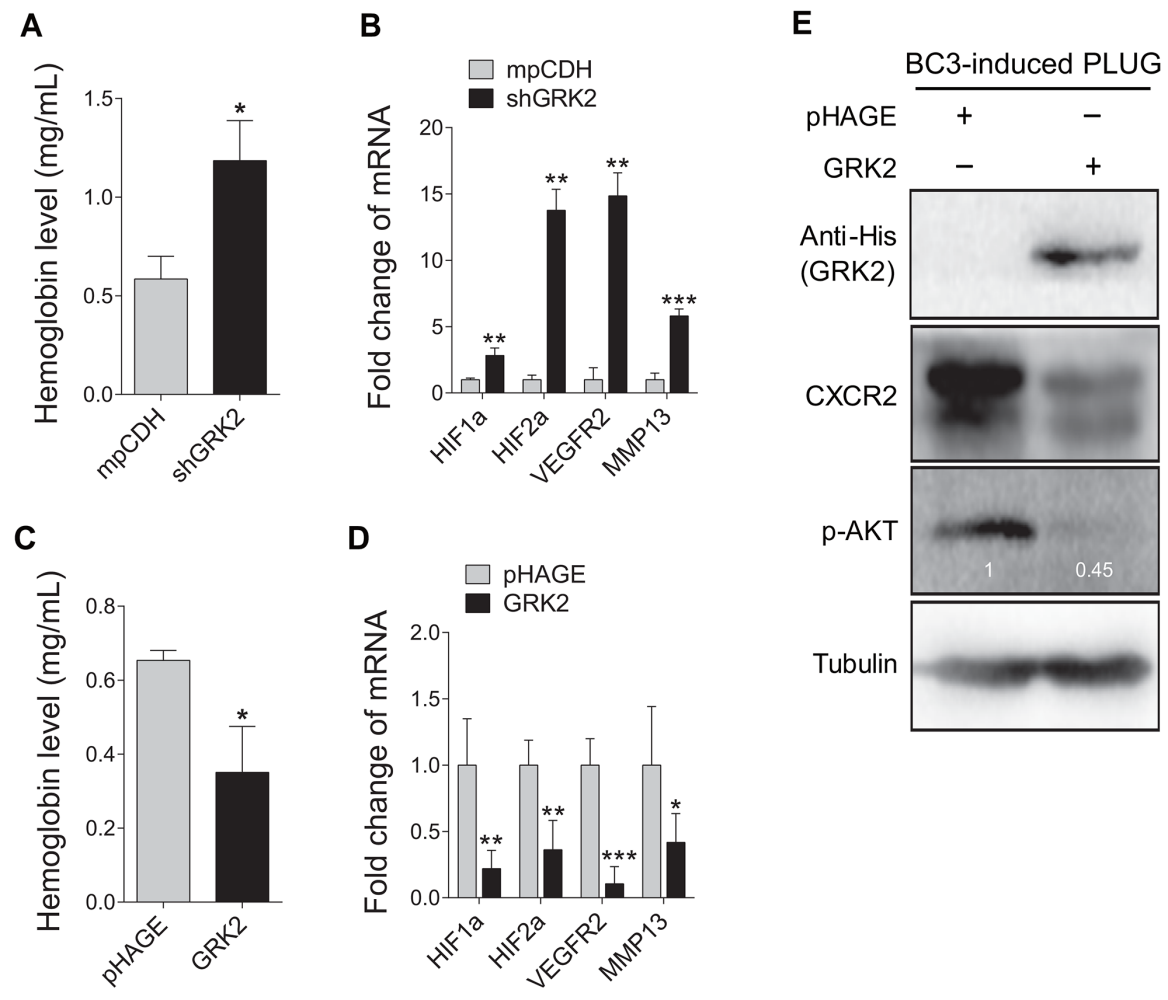
GRK2 inhibits angiogenesis induced by KSHV **A.** Suppression of GRK2 promoted KSHV-induced angiogenesis in nude mice. BC3 cells transduced with lentivirus-mediated a mixture of short hairpin RNAs targeting GRK2 (shGRK2) or the control (mpCDH) were examined for their pro-angiogenic effects in nude mice by the Matrigel plug assay. The hemoglobin level of the Matrigel plugs was determined with O.D. value at 540 nm in BC3 cells-induced tumor tissues. Data represented mean ± SD, each group with five tumors (*n*=5). Three independent experiments were performed and similar results were obtained. *P < 0.05 and ***P < 0.001 for Student's *t*-test. **B.** The transcriptional levels of HIF1α, HIF2α, VEGFR2, and MMP13 mRNAs were measured by RT-qPCR in the Matrigel plugs treated as in (A). **P < 0.01 and ***P < 0.001 for Student's *t*-test. **C.** BC3 cells transduced with lentivirus-GRK2 (GRK2) or the control (pHAGE) were examined for their pro-angiogenic effects in nude mice by the Matrigel plug assay. The hemoglobin level of the Matrigel plugs was determined with O.D. value at 540 nm. Data represented mean ± SD, each group with five tumors (*n*=5). Three independent experiments were performed and similar results were obtained. *P < 0.05 for Student's *t*-test. **D.** The mRNA expression levels of HIF1α, HIF2α, VEGFR2, and MMP13 were measured by RT-qPCR in the Matrigel plugs treated as in (C). *P < 0.05, **P < 0.01 and ***P < 0.001 for Student's *t*-test. **E.** Western blotting analysis of CXCR2 expression and phosphorylation levels of AKT in the Matrigel plugs treated as in (C). The antibody against His-tag was used to detect the exogenous expression of GRK2.

To determine the role of GRK2 in miR-K3-induced angiogenesis, Matrigel plug assays were performed. We found that knockdown of GRK2 not only markedly enhanced KSHV-induced angiogenesis (Figure 5A), but also increased transcriptional expression levels of essential angiogenesis-related genes. On the contrary, overexpression of GRK2 in BC3 cells impaired the angiogenic capability of KSHV in plug assay. Consistently, overexpression of GRK2 with lentivirus transduction dramatically reduced the levels of CXCR2 protein and phosphorylated AKT (Figure 5E). These data suggest that GRK2 targeted by miR-K3 negatively regulates KSHV latency and angiogenesis.

### Activation of CXCR2/AKT signaling contributes to miR-K3-induced KSHV latency and angiogenesis

**Figure 6:**
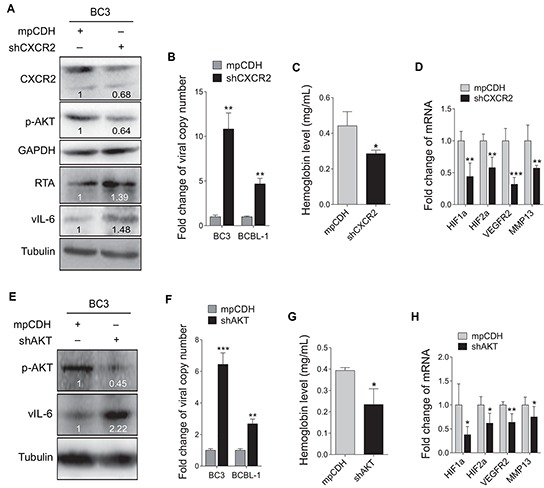
Inhibition of CXCR2/AKT pathway disrupts KSHV latency and suppresses angiogenesis **A.** Western blotting analysis of the expression levels of CXCR2, phosphorylated AKT, RTA and vIL-6 in BC3 cells transduced with lentivirus-mediated a mixture of short hairpin RNAs targeting CXCR2 (shCXCR2) or the control (mpCDH). **B.** Real-time DNA-PCR was performed to detect the viral genome copy number in the supernatants of BC3 and BCBL-1 cells treated as in (A). ** *P* < 0.01 for Student's *t*-test. **C.** BC3 cells transduced with lentivirus-mediated a mixture of short hairpin RNAs targeting CXCR2 (shCXCR2) or the control (mpCDH) were examined for pro-angiogenic effects in nude mice by the Matrigel plug assay. The hemoglobin level of the Matrigel plugs was determined with O.D. value at 540 nm. Data represented mean ± SD, each group with five tumors (*n*=5). Three independent experiments were performed and similar results were obtained. *P < 0.05 for Student's t-test. **D.** The mRNA expression levels of HIF1α, HIF2α, VEGFR2 and MMP13 were measured using RT-qPCR in tumor tissues from the Matrigel plugs treated as in (C). **P < 0.01 and ***P < 0.001 for Student's *t*-test. **E.** Western blotting analysis of phosphorylated AKT and vIL-6 in BC3 cells transduced with lentivirus-mediated a mixture of short hairpin RNAs targeting AKT (shAKT) or the control (mpCDH). **F.** Real-time DNA-PCR was performed to detect the viral genome copy number in the supernatant of BC3 cells treated as in (E). ** *P* < 0.01 and *** *P* < 0.001 for Student's *t*-test. **G.** The Matrigel plug assay was performed to examine the pro-angiogenic effect of BC3 cells transduced with shAKT (shAKT) or the control (mpCDH) in nude mice. The hemoglobin level of the Matrigel plugs was determined with O.D. value at 540 nm. Data represented mean ± SD, each group with five tumors (*n*=5). Three independent experiments were performed and similar results were obtained. *P < 0.05 for Student's t-test. **H.** RT-qPCR was performed to detect the mRNA expression levels of HIF1α, HIF2α, VEGFR2 and MMP13 in tumor tissues from the Matrigel plugs treated as in (G). *P < 0.05 and **P < 0.01 for Student's *t*-test.

To determine whether the CXCR2/AKT signaling was involved in the promotion of KSHV latency and angiogenesis by miR-K3, BC3 cells were firstly transduced with a mixture of lentivirus-mediated short hairpin RNAs targeting CXCR2 (shCXCR2). As shown in Figure 6A, knockdown of CXCR2 decreased the phosphorylated AKT level, and elevated the protein levels of RTA and vIL-6. Consistently, inhibition of CXCR2 increased the production of viral particles (Figure 6B). To determine the role of CXCR2 in KSHV-induced angiogenesis, Matrigel plug assays were performed. We found that knockdown of CXCR2 inhibited BC3 cells-induced angiogenesis (Figure 6C). qPCR showed that knockdown of CXCR2 also decreased mRNA expression of cellular genes associated with angiogenesis and invasion including HIF1α, HIF2α, VEGFR2 and MMP13 in plug tissues induced by BC3 cells (Figure 6D).

Because AKT is a downstream mediator of CXCR2 and plays a critical role in miR-K3 induction of cell invasion [40], we next examined the effect of AKT on KSHV latency and angiogenesis. Knockdown of AKT in BC3 increased the protein expression of vIL-6, and the production of viral particles (Figure 6E and 6F). Similarly, knockdown of AKT in BC3 cells inhibited KSHV-induced hemoglobin levels (Figure 6G), and decreased transcripts of HIF1α, HIF2α, VEGFR2 and MMP13 genes in BC3 cells-induced plug tissues (Figure 6H). Together these data suggest that the CXCR2/AKT pathway is involved in miR-K3-induced KSHV latency and angiogenesis.

### Deletion of MiR-K3 from KSHV genome disrupts KSHV latency and impairs KSHV-induced angiogenesis

**Figure 7:**
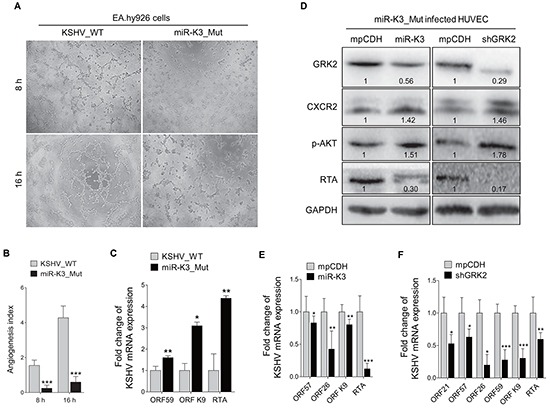
Deletion of miR-K3 from the KSHV genome reactivates KSHV lytic replication but inhibits angiogenesis **A.** Matrigel assay analysis of microtubule formation was performed in EA.hy926 cells, which were incubated with the supernatants from stable iSLK-BAC16 (KSHV_WT) or BAC16ΔmiR-K3 (miR-K3_Mut) cells, as described in the ‘Materials and Methods’ section. The photographs of microtubules were taken at 8 and 16 h post-seeding (original magnification, × 100). **B.** Quantification of results in (A). Data represented mean ± SD. ***P < 0.001 for Student's *t*-test. **C.** The mRNA expression levels of KSHV ORF59, ORF-K9 and RTA in HUVECs infected with BAC16 KSHV wild type virus (KSHV_WT) or BAC16 miR-K3 deletion mutant virus (miR-K3_Mut) were detected by RT-qPCR. *P < 0.05 and ** *P* < 0.01 for Student's *t*-test. **D.** Western blotting analysis of GRK2, CXCR2, phosphorylated AKT and RTA proteins in HUVECs transduced with lentivirus-miR-K3 (miR-K3), lentivirus-mediated shGRK2 (shGRK2) or their respective control (mpCDH) after infection with BAC16 KSHV miR-K3 deletion mutant virus. **E.** The mRNA expression levels of ORF57, ORF26, ORF-K9 and RTA in HUVEC treated as in (D; left panel) were determined by RT-qPCR. *P < 0.05, **P < 0.01 and ***P < 0.001 for Student's *t*-test. **F.** The mRNA expression levels of ORF21, ORF57, ORF26, ORF59, ORF-K9 and RTA in HUVEC treated as in (D; right panel) were determined by RT-qPCR. *P < 0.05, **P < 0.01 and ***P < 0.001 for Student's *t*-test.

The BAC16 miR-K3_Mut virus with a deletion of miR-K3 from KSHV genome was further used to investigate the role of miR-K3 in KSHV lifecycle and angiogenesis. In the tube formation assay, deletion of miR-K3 significantly impaired the angiogenic effect of KSHV in HUVECs (Figure 7A and 7B). Consistently, deletion of miR-K3 also decreased the amount of secreted VEGFA protein in the supernatants of cells (Supplementary Figure S2A). qPCR showed that KSHV lytic transcripts ORF59, ORF-K9 and RTA were significantly increased in HUVECs infected with BAC16 miR-K3_Mut virus compared to those infected with wide type KSHV (Figure 7C). Further, expression of miR-K3 in miR-K3_Mut virus-infected HUVECs reversed the above phenotypes, and markedly inhibited the expression of KSHV lytic genes, decreased the protein level of GRK2, and increased the protein level of CXCR2 (Figure 7D and 7E). On the other hand, knockdown of GRK2 in miR-K3_Mut virus-infected HUVECs dramatically suppressed the mRNA and protein expression of KSHV lytic genes (Figure 7D and 7F). We have also detected increased level of secreted VEGFA protein in the supernatants and enhanced angiogenesis in miR-K3_mut-infected HUVEC following overexpression of miR-K3 or knock-down of GRK2 (Supplementary Figure S2B, S2C and Supplementary Figure S3). Collectively, these data suggest that by targeting GRK2, miR-K3 promotes KSHV latency and angiogenesis by activating the CXCR2/AKT pathway.

## DISCUSSION

KSHV lifecycle consists of latent and lytic replication phases. Following primary infection, KSHV establishes latency, which allows the virus to evade host immune surveillance. Since most tumor cells in KS, PEL, and MCD are latently infected by KSHV, latent infection likely facilitates KSHV-induced malignancy and pathogenesis. Meanwhile, a small number of KSHV-infected tumor cells can be reactivated into lytic replication leading to the production of infectious virions, which can be spread to the uninfected cells, as well as promote the initiation of KS tumors through an autocrine and paracrine effect. Due to the distinct roles of these two phases in KSHV-related malignancies, better understanding the mechanisms of KSHV latency and reactivation might be crucial to unravel KSHV-induced pathogenesis.

KS pathogenesis usually depends on the fine balance between latency and viral lytic replication. This balance is controlled by RTA and regulated by latent gene products, such as LANA, cellular signaling pathways, and cellular and/or viral miRNAs [52]. As a KSHV-encoded miRNA, miR-K3 is highly expressed in KS tumors [41], and in KSHV-infected PEL cell lines [53, 54], indicating its potential role in viral lifecycle and its oncogenic potential. So far, the validated targets of miR-K3 included NFIB, MYB, C/EBPα, Ets-1 and GRK2 [19, 39, 40]. Among them, GRK2 was recently identified by our group. We have shown that GRK2 mediates miR-K3-induced cell migration and invasion, implying that it might play a key role in KSHV-induced tumor dissemination and invasion. In the current study, we have shown pivotal roles of miR-K3, its target, GRK2, and the downstream CXCR2/AKT pathway in viral lifecycle and angiogenesis. miR-K3 directly targets GRK2 to promote KSHV latency and angiogenesis by activating the CXCR2/AKT pathway. This novel finding has broadened the regulatory network of KSHV miRNAs and their targets.

GRK2 not only functions to modulate G-protein-coupled receptor signaling, but also exhibits very diverse functions including control of cell motility and growth, and modulation of inflammatory responses. Emerging evidences suggest that GRK2 modulates multiple cellular responses in various physiological contexts by either phosphorylating non-receptor substrates or by interacting with signaling molecules. GRK2 also regulates developmental and tumoral vascularization. Downregulation of GRK2 would be a relevant event in the angiogenic switch triggered by tumor cells by favoring a permissive microenvironment for tumor progression [55]. Notably, GRK2 has been reported to interact directly with AKT to reduce NO production in endothelial cells [56]. A similar phenomenon has been shown in our previous study that GRK2 binding to AKT is involved in the migration and invasion of endothelial cells [40]. Consistently, in the current study, our co-immunoprecipitation results also showed that GRK2 directly interacts with AKT in KSHV-infected B cells. Our findings indicate that KSHV maintains latency and induces angiogenesis in diverse cell types by inhibiting GRK2 to activate AKT.

It has been reported that interleukin-8 (IL-8), a member of the neutrophil-specific C-X-C subfamily of chemokines, acts on endothelial cells via binding onto either CXCR1 or CXCR2 to promote invasion and angiogenesis [57]. IL-8 and its receptor CXCR2 are significantly upregulated in the tumors and tumor microenvironment in many cancers including colorectal and pancreatic cancers. Inhibition of CXCR2 dramatically decreased angiogenesis and tumor growth by suppressing AKT signaling [58, 59]. Further, by activating the CXCR2 and AKT signaling, HIV-1 matrix protein p17 promotes endothelial dysfunction and angiogenesis in AIDS-related vascular diseases [60]. We have shown that blockade of CXCR2 or AKT by short-hairpin RNA knockdown attenuates PEL-induced angiogenesis and decreases pro-angiogenic factors. The AKT pathway is constitutively activated in various human malignancies. As expected, AKT signaling is also activated during KSHV *de novo* infection [61]. To date, there are four KSHV viral proteins that are known to impinge upon AKT signaling to exert their corresponding functions. They are K1, viral G protein-coupled receptor (vGPCR), vIL-6, and ORF45 [62]. In addition, inhibition of the AKT pathway enhances KSHV lytic replication and facilitates reactivation from latency, suggesting that activation of the AKT pathway contributes to the maintenance of viral latency and promotes tumorigenesis [63].

In agreement with these results, we have demonstrated that knockdown of AKT disrupts KSHV latency by inducing viral lytic replication. Our results are therefore consistent with a hypothesis that activation of the AKT pathway promotes viral latency by negatively regulating viral lytic replication. Although constitutive activation of AKT maintains KSHV latency in PEL cells, the underlying mechanism remains unclear [63]. Importantly, in this study we have shown that in KSHV-infected B lymphoma cells, abundant miR-K3 expressed by KSHV might directly target GRK2 and inhibit its expression, resulting in downregulation of GRK2, increase of CXCR2 and activation of AKT, which lead to the promotion KSHV latency. Our novel findings provide an explanation for the constitutive activation of AKT and its possible functions in KSHV-infected B lymphoma cells and endothelial cells.

**Figure 8:**
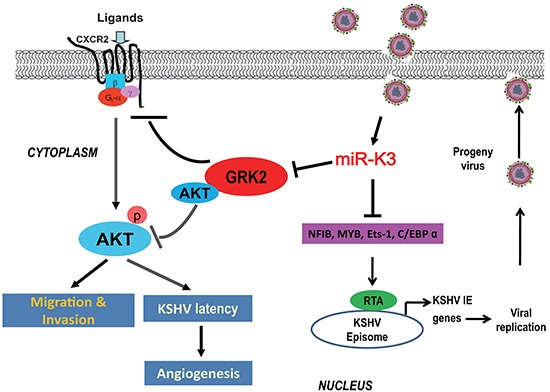
A model for the effect of miR-K3 on inhibition of KSHV lytic replication and promotion of KSHV-induced angiogenesis and invasion Expression of miR-K3 downregulates NFIB, MYB, C/EBPα and Ets-1, which activates the promoter activity of KSHV immediate-early (IE) gene, Rta, resulting in inhibition of viral lytic replication. miR-K3 also directly targets GRK2 promoting cell migration and invasion, and KSHV latency and angiogenesis by activating the CXCR2/AKT pathway.

In conclusion, our studies provide significant evidence that besides migration and invasion, miR-K3 also enhances KSHV latency and angiogenesis through activating the CXCR2/AKT pathway by targeting GRK2. Since miR-K3 has multiply functions in regulating KSHV infection and pathogenesis via multiple targets (Figure 8), miR-K3 and its regulated proteins and pathways may represent novel therapeutic targets for KSHV-induced malignancies.

## MATERIALS AND METHODS

### Cell culture and recombinant KSHV virus

The KSHV-positive and EBV-negative PEL cell lines BC-3 and BCBL-1, and KSHV-negative and EBV-negative B lymphocyte lines DG75, Loukes and BJAB cells were maintained in RPMI-1640 containing 10% heat-inactivated fetal bovine serum (FBS), 2 mmol/L of L-glutamine, 100 U/ml of penicillin, and 100 μg/mL of streptomycin at 37°C in a humidified, 5% CO_2_ atmosphere. HEK293T and EA.hy926 cells were grown in Dulbecco's modified Eagle's medium (DMEM) with 10% FBS. EA.hy926 is an immortalized cell line obtained from fusion of primary human umbilical vein cells and the A549 human lung adenocarcinoma cell line, which has the characteristics of vascular endothelial cells [64]. Primary human umbilical vein endothelial cells (HUVECs) were isolated from the interior of the umbilical vein of human umbilical cords by digestion with collagenase (Sigma, St. Louis, MO, USA) as described [65]. HUVECs were cultured in complete EBM-2 culture media (LONZA, Allendale, NJ, USA) and used between passage 3 and 6. Wild type recombinant KSHV BAC16 and a KSHV mutant with miR-K3 deleted, BAC16ΔmiR-K3, were previously described [39, 66].

### Plasmid

The recombinant lentiviral plasmid pHAGE-GRK2, the microRNA lentiviral expressing plasmid miR-K3, miR-K3 sponge lentiviral plasmid, and the short hairpin RNA (shRNA) expressing lentiviral vectors including shGRK2, shCXCR2 and shAKT were previously described [40]. In this study, the control of pHAGE-GRK2 was named as pHAGE and the controls for the expression constructs of miR-K3 and shRNAs were a modified lentiviral pCDH (mpCDH for short); while the control for the miR-K3 sponge was designed as pCDH.

### Production and transduction of lentivirus

To obtain the recombinant lentivirus, the virus-packaging cells HEK293T were seeded for 24 h and later then co-transfected with lentiviral plasmids, packaging vector psPAX2 and envelope vector pMD2.G as previously described [67]. The virus containing supernatants were collected 48 h after transfection.

### Antibodies and reagents

Anti-KSHV vIL-6 rabbit polyclonal antibody (PAb) was purchased from Advanced Biotechnologies Inc. (Columbia, MD, USA). Anti-KSHV ORF65 mouse monoclonal antibody (MAb) was used to detect KSHV capsids [68]. Anti-KSHV RTA peptide antibodies were generated by immunization of rabbits with RTA peptide as previously described [69]. Anti-phospho-AKT (Ser473) mouse MAb, anti-AKT rabbit PAb, anti-GRK2 rabbit PAb, anti-CD31 mouse MAb and anti-His rabbit MAb were obtained from Cell Signaling Technologies (Beverly, MA, USA). Anti-CXCR2 rabbit PAb, anti-α-tubulin and GAPDH mouse MAbs, and horseradish peroxidase (HRP)-conjugated goat anti-mouse and anti-rabbit IgG were purchased from Santa Cruz Biotechnology (Santa Cruz, CA, USA). Anti-smooth muscle actin (SMA) rabbit PAb were obtained from AbbiotecTM (San Diego, CA, USA). 12-*O*-tetradecanoylphorbol-13-acetate (TPA; 20 ng/mL), doxycycline hyclate (1 μg/ml) and sodium butyrate (1 mM) were obtained from Sigma (St. Louis, MO, USA). 4′, 6′-diamidino-2-phenylindole (DAPI) was purchased from Beyotime Institute of Biotechnology (Nantong, China).

### Real-time quantitative reverse transcription-PCR (RT-qPCR)

Total RNA was isolated from cells by Trizol reagent (Invitrogen). RT-qPCR was performed using SYBR *Premix Ex Taq*™ Kit (TaKaRa Biotechnology Co.Ltd., Dalian, China) according to the manufacturer's instructions. The quantification of miRNAs was performed by stem-loop RT-qPCR as previously described [15]. The sequences of specific primers of RT-qPCR for KSHV lytic genes and cellular angiogenic factors were listed in Table 1 and Table 2, respectively.

**Table 1:** Primers for RT-qPCR detection of KSHV genes

Target	Application	Primer
ORF26	RT-qPCR	F: 5′-AGC CGA AAG GAT TCC ACC AT-3′
		R: 5′-GCT GCG GCA CGA CCA T-3′
ORF21	RT-qPCR	F: 5′-CGT AGC CGA CGC GGA TAA-3′
		R: 5′-TGC CTG TAG ATT TCG GTC CAC-3′
ORF57	RT-qPCR	F: 5′-TGG CGA GGT CAA GCT TAA CTT C-3′
		R: 5′-CCC CTG GCC TGT AGT ATT CCA-3′
ORF59	RT-qPCR	F: 5′-TTG GCA CTC CAA CGA AAT ATT AGA A-3′
		R: 5′-CGG GAA CCT TTT GCG AAG A-3′
ORF-K9	RT-qPCR	F: 5′-GTC TCT GCG CCA TTC AAA AC-3′
		R: 5′-CCG GAC ACG ACA ACT AAG AA-3′
LANA	RT-qPCR	F: 5′-CCG AGG ACG AAA TGG AAG TG-3′
		R: 5′-GGT GAT GTT CTG AGT ACA TAG CGG-3′
RTA	RT-qPCR	F: 5′-GCG CAA GAT GAC AAG GGT AAG-3′
		R: 5′-CGA GAG GCC GAC GAA GC-3′

F, forward; R, reverse

**Table 2:** Primers for RT-qPCR detection of cellular angiogenic factors and house-keeping genes

Target	Application	Primer
HIF1α	RT-qPCR	F: 5′-CAA GAT CTC GGC GAA GCA A-3′
		R: 5′-GGT GAG CCT CAT AAC AGA AGC TTT-3′
HIF2α	RT-qPCR	F: 5′-CGG AGG TGT TCT ATG AGC TGG-3′
		R: 5′-AGC TTG TGT GTT CGC AGG AA-3′
VEGFR2	RT-qPCR	F: 5′-TGG GAA CCG GAA CCT CAC TAT C-3′
		R: 5′-GTC TTT TCC TGG GCA CCT TCT ATT-3′
MMP13	RT-qPCR	F: 5′-TGG AAG GAT GCC TTT TTT TCT C-3′
		R: 5′-CAC CCT CCC CAA GTA TCA ATA GG-3′
β-actin	RT-qPCR	F: 5′-TTG CCG ACA GGA TGC AGA AGG A-3′
		R: 5′-AGG TGG ACA GCG AGG CCA GGA T-3′
GAPDH	RT-qPCR	F: 5′-GAA GGT GAA GGT CGG AGT C-3′
		R: 5′-GAA GAT GGT GAT GGG ATT TCC-3′

F, forward; R, reverse

### Western blotting analysis

Western blotting was performed as previously described [70]. Differences in protein expression were determined by densitometry analysis using Scion Image software (Scion Corporation, Frederick, MD, USA). Data shown were repeated at least three times to confirm the results.

### Immunofluorescence assay (IFA)

Immunofluorescence assay was performed as previously described [71]. Images were observed and recorded with a Zeiss Axiovert 200 M epifluorescence microscope (Carl Zeiss, Inc.).

### Real-time DNA-PCR analysis for viral genome copy number

To examine the production of viral progeny, real-time DNA-PCR analysis for viral genome copy number was performed as previously described [71].

### Production of BAC16 virus stock

Production of KSHV BAC16 virus was performed as previously described [66]. Briefly, stable iSLK-BAC16 or BAC16ΔmiR-K3 cells were treated with both doxycycline (1 μg/ml) and sodium butyrate (1 mM). Two days later, the old medium was removed and replaced with maintaining medium. The cells were maintained for another two or three days, and cell supernatant was collected, and cleared of cells and debris by centrifugation (3,000 rpm for 10 min at room temperature) and filtration (0.45 μm). Virus particles were pelleted by ultracentrifugation from the cell supernatant through 20% sucrose cushion (24,000 rpm for 3 h at 4°C) using an SW32 Ti rotor. The supernatant was then discarded and the virus pellet was re-suspended in a desired volume.

### Transfection and luciferase reporter assay

Transfections were performed with Lipofectamine™ 2000 (Invitrogen, Inc., Carlsbad, CA, USA) according to the manufacturer's instructions. For luciferase reporter assay, cells were harvested after transfection for 48 h. Renilla vector pRL-TK (Promega, Madison, WI, USA) was used as an internal control and the relative luciferase activity was assayed using Promega dual-luciferase reporter assay system.

### Matrigel plug assay for angiogenesis in nude mice

All animal care and use protocols were performed in accordance with the Regulations for the Administration of Affairs Concerning Experimental Animals approved by the State Council of People's Republic of China. The animal experiments were approved by the Institutional Animal Care and Use Committee of Nanjing Medical University. Female athymic BALB/c nu/nu mice at 3-4 weeks old (Shanghai Slac Laboratory Animal Center, Shanghai, China) were maintained under pathogen-free conditions. Cell aliquots were mixed with High Concentration Matrigel (BD Biosciences, Bedford, MA, USA), and the mixture was immediately injected subcutaneously into the right flanks of nude mice. The hemoglobin content of the Matrigel was determined by Drabkin's reagent kit (Sigma-Aldrich) according to the manufacturer's instructions. The final hemoglobin concentration was calculated using a standard calibration curve after spectrophotometric analysis at 540 nm [67, 72, 73].

### Immunohistochemistry (IHC)

Informative sections of formalin-fixed and paraffin-embedded tumor samples from nude mice were immunostained with the indicated antibodies, as previously described [74].

### Tubule formation assay

The microtubule formation assay was performed on 96-well plates coated with 50 μl of Matrigel (BD Biosciences, Bedford, MA, USA). Stable iSLK-BAC16 (KSHV_WT) or BAC16ΔmiR-K3 (miR-K3_mut) cells were treated with both doxycycline (1 μg/ml) and sodium butyrate (1 mM). Two days later, the old medium was removed and replaced with maintaining medium. The cells were cultured for another two or three days, and cell supernatant was collected and added to the EA.hy926 cells pellet. The EA.hy926 cells were resuspended and seeded at 1 × 10^4^ cells per well in 96-well plate. Tubule formation was quantified by counting the number of branching points and measuring the total length of the capillary tubes in at least five images using NIH ImageJ software [67, 72, 75]. The angiogenesis index was calculated according to the formula described elsewhere [76].

### Co-immunoprecipitation

Using total cell lysates, immunoprecipitation was performed as described previously [40].

### ELISA

The amounts of VEGFA in culture supernatants were measured by ELISA kit (R&D Systems). Absorbance at 450 nm was measured using an ELISA microplate reader.

### Statistical analysis

Quantitative data were presented as mean ± SD. Two-sided Student's *t*-test was used to determine the significance between different treatment groups. *P* < 0.05 was considered statistically significant.

## SUPPLEMENTARY FIGURES


