# Supplementary material for: A KSHV microRNA enhances viral latency and induces angiogenesis by targeting GRK2 to activate the CXCR2/AKT pathway
Source: Oncotarget. 2016 Apr 5;7(22):32286–305. doi: 10.18632/oncotarget.8591 (PMC5078013; doi:10.18632/oncotarget.8591)
Supplement: Supplementary file 1 [file oncotarget-07-32286-s001.pdf]

A KSHV microRNA enhances viral latency and induces angiogenesis by targeting GRK2 to activate the CXCR2/AKT pathway

SUPPLEMENTARY FIGURES

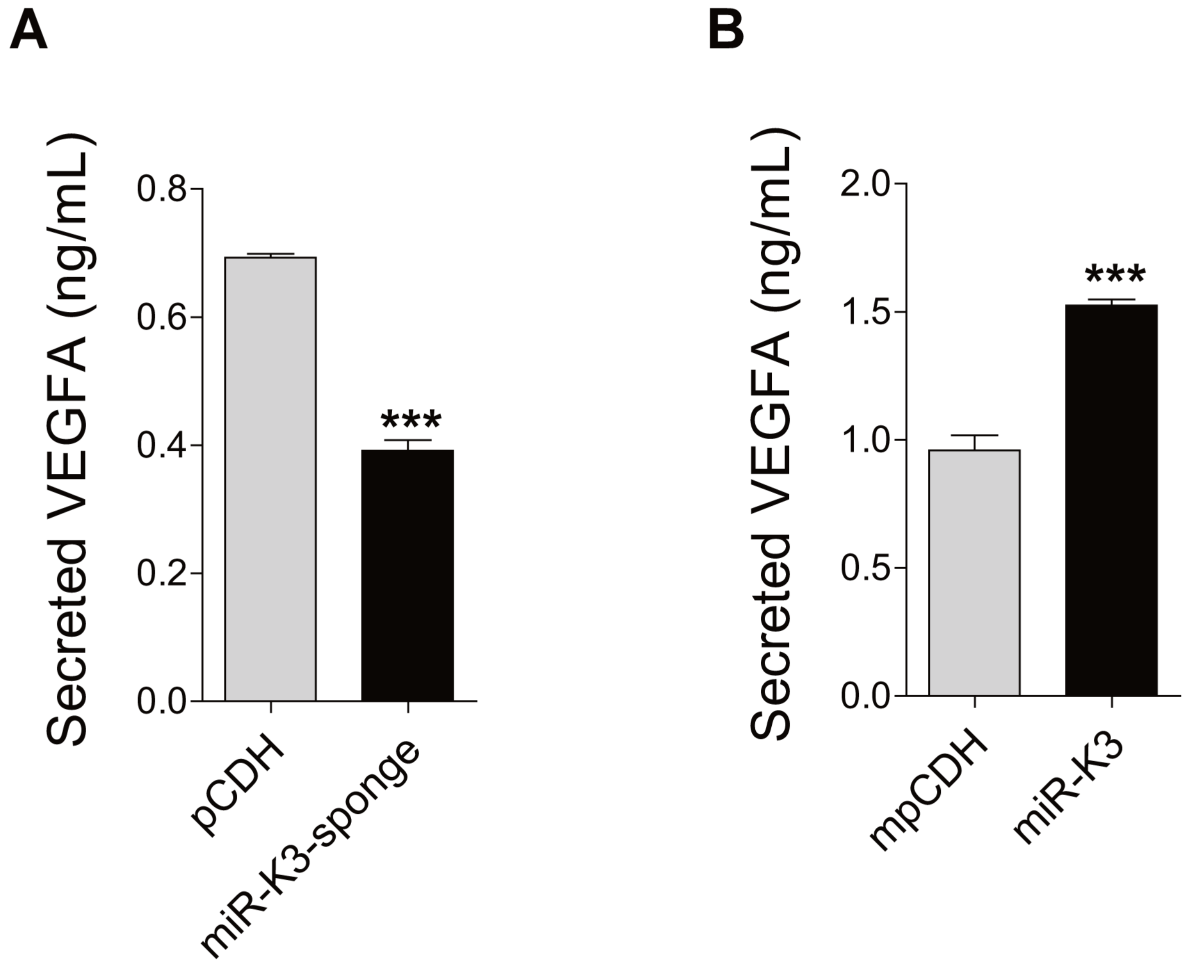

Supplementary Figure S1: KSHV-encoded miR-K3 induces VEGFA secretion by BC3 cells in vitro. A. Enzyme-linked immunosorbent assay (ELISA) measurement of secreted VEGFA in the supernatants from BC3 cells transduced with miR-K3 sponge (miR-K3 sponge) or the control (pCDH) for 48 h, respectively. ***$P < 0.001$ for Student's *t*-test. **B.** Enzyme-linked immunosorbent assay (ELISA) measurement of secreted VEGFA in the supernatants from BC3 cells transduced with lentivirus-miR-K3 (miR-K3) or the control lentivirus (mpCDH) for 48 h, respectively. ***$P < 0.001$ for Student's t-test.

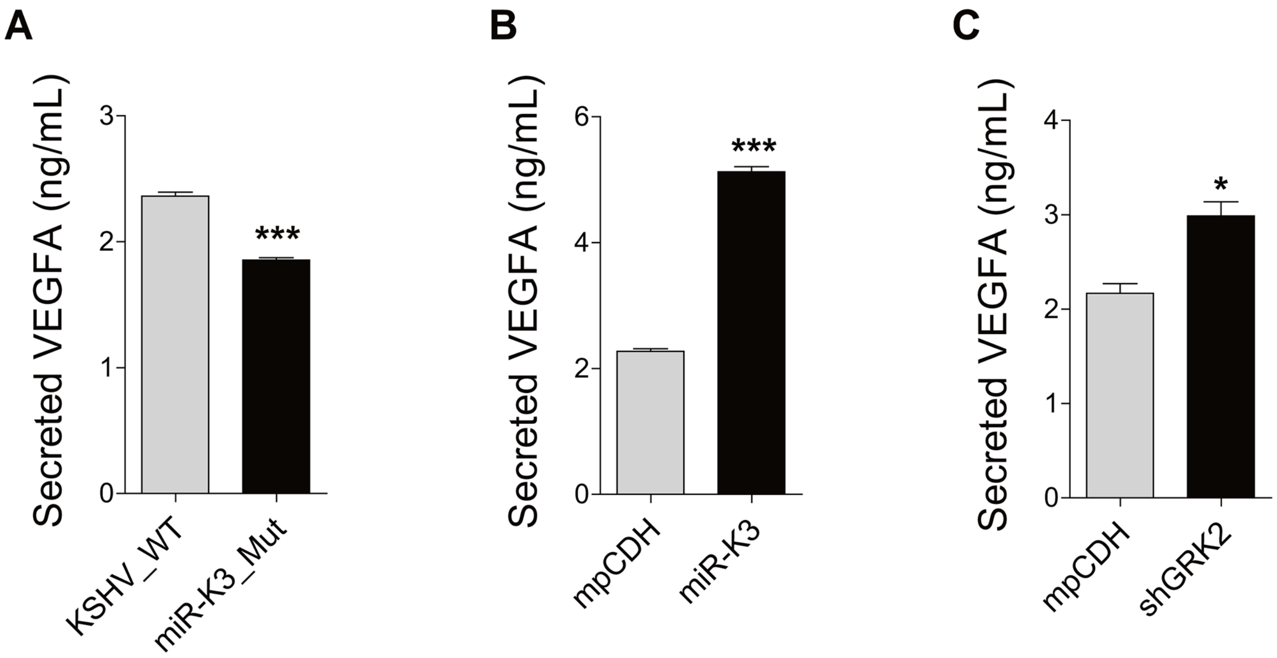

Supplementary Figure S2: Deletion of miR-K3 from KSHV genome reduces VEGFA secretion by HUVEC in vitro. **A.** Enzyme-linked immunosorbent assay (ELISA) measurement of secreted VEGFA in the supernatants from HUVECs infected with BAC16 KSHV wild type virus (KSHV_WT) or BAC16 miR-K3 deletion mutant virus (miR-K3_Mut). ***$P < 0.001$ for Student's t-test. **B.** Enzyme-linked immunosorbent assay (ELISA) measurement of secreted VEGFA in the supernatants from HUVECs transduced with lentivirus-miR-K3 (miR-K3) or its respective control (mpCDH) after infection with BAC16 KSHV miR-K3 deletion mutant virus. ***$P < 0.001$ for Student's t-test. **C.** Enzyme-linked immunosorbent assay (ELISA) measurement of secreted VEGFA in the supernatants from HUVECs transduced with lentivirus-mediated shGRK2 (shGRK2) or its respective control (mpCDH) after infection with BAC16 KSHV miR-K3 deletion mutant virus. *$P < 0.05$ for Student's *t*-test.

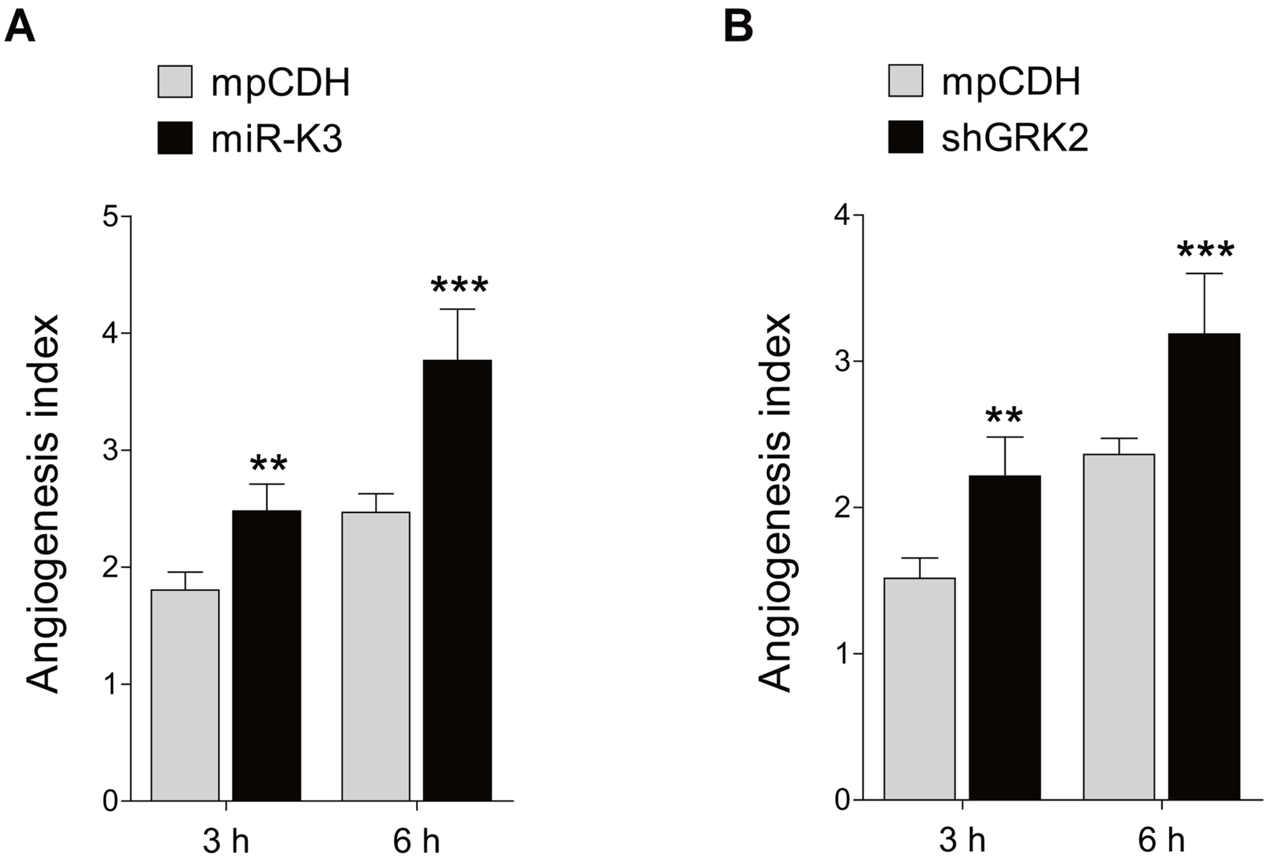

Supplementary Figure S3: MiR-K3/GRK2 pathway is necessary for KSHV-induced angiogenesis. A. Microtubule formation assay for HUVECs transduced with lentivirus-miR-K3 (miR-K3) or its respective control (mpCDH) after infection with BAC16 KSHV miR-K3 deletion mutant virus. $^{**}P < 0.01$, and $^{***}P < 0.001$ for Student's t-test. **B.** Microtubule formation assay for HUVECs transduced with lentivirus-mediated shGRK2 (shGRK2) or its respective control (mpCDH) after infection with BAC16 KSHV miR-K3 deletion mutant virus. $^{**}P < 0.01$, and $^{***}P < 0.001$ for Student's t-test.
